# Measures of General Intelligence and Risk for Alcohol Use Disorder

**DOI:** 10.1001/jamapsychiatry.2025.2689

**Published:** 2025-10-01

**Authors:** Andrea Johansson Capusan, Christal N. Davis, Emelie Thern, Jürgen Rehm, Joel Gelernter, Henry R. Kranzler, Markus Heilig

**Affiliations:** 1 Center for Social and Affective Neuroscience, Department of Biomedical and Clinical Sciences, Linköping University, Linköping, Sweden; 2 Mental Illness Research, Education, and Clinical Center, Crescenz VAMC, Philadelphia, Pennsylvania; 3 Center for Studies of Addiction, Department of Psychiatry, University of Pennsylvania Perelman School of Medicine, Philadelphia; 4 Institute of Environmental Medicine, Karolinska Institute, Stockholm, Sweden; 5 Institute for Mental Health Policy Research & Campbell Family Mental Health Research Institute, Centre for Addiction and Mental Health, Toronto, Ontario, Canada; 6 Dalla Lana School of Public Health, Institute of Health Policy, Management and Evaluation, Department of Psychiatry, University of Toronto, Toronto, Ontario, Canada; 7 Zentrum für Interdisziplinäre Suchtforschung der Universität Hamburg, Universitätsklinikum Hamburg-Eppendorf, Hamburg, Germany; 8 Department of Psychiatry, Yale University School of Medicine, New Haven, Connecticut; 9 VA Connecticut Healthcare System, West Haven, Connecticut

## Abstract

**Importance:**

Associations among general intelligence (IQ), educational attainment (EA), and alcohol use disorder (AUD) are not well understood.

**Objective:**

To examine the relationship between IQ, EA, and AUD risk.

**Design, Setting, and Participants:**

The association between IQ and AUD risk was examined in a Swedish national conscription cohort. Potential causality was explored using mendelian randomization (MR) analyses, and the association of polygenic scores (PGS) for cognitive performance with AUD diagnosis was assessed. Participant data were obtained from cross-linked Swedish national registers, genome-wide association study (GWAS) summary statistics, and the US Yale-Penn cohort.

**Exposures:**

IQ and genetic variants associated with cognitive performance.

**Main Outcomes and Measures:**

Hazard ratios (HRs; time-to-event analyses) or odds ratios (ORs) for AUD.

**Results:**

Included in this study was a national cohort of 645 488 males, born between 1950 and 1962, from the Swedish Military Conscription Register, of whom 573 855 individuals were included in this analysis. All individuals were aged 18 years at IQ assessment with no substance use disorder diagnosis at conscription, and mean (SD) follow-up time (SD) was 60.5 (7.9) years. Summary statistics from GWAS of cognitive performance (n = 257 481) and AUD (total = 753 248; cases = 113 325) in individuals of European-like genetic ancestry (EUR), with FinnGen AUD GWAS as a replication sample (total = 500 348; cases = 20 597), were used for MR analyses. PGS analyses were conducted using the data of EUR individuals from the Yale-Penn cohort (n = 5424). IQ at age 18 years was inversely associated with AUD risk in Swedish males (adjusted HR, 1.43; 95% CI, 1.40-1.47; *P* < .001), adjusting for parental substance use disorder, probands’ psychiatric disorders, socioeconomic factors, and birth year strata. MR analyses suggested a causal relationship between lower cognitive performance and AUD risk (β [SE], 0.11 [0.02]; *P* = 2.6 × 10^−12^). The mediating role of EA differed between national contexts. Higher cognitive performance PGS were associated with reduced odds of AUD in Yale-Penn participants (OR, 0.83; 95% CI, 0.78-0.89).

**Conclusions and Relevance:**

IQ and cognitive performance have a significant but context-dependent association with AUD risk, highlighting the need for a better understanding of the interplay among genetic factors, cognitive traits, and sociocultural influences on AUD susceptibility.

## Key Points

**Question:**

Is there an association between IQ and risk for alcohol use disorder, and if so, what is the nature of this association?

**Findings:**

In a male Swedish cohort including 573 855 participants, IQ at age 18 years was associated with subsequent alcohol use disorder risk. Mendelian randomization analyses suggest a causal association, albeit with context-dependent differences; genetic liability for cognitive performance also predicted alcohol use disorder in a US-based sample.

**Meaning:**

Results suggest that there was a clear impact of genetic liability for cognitive performance on alcohol disorder risk, but the association varies based on the sociocultural context.

## Teaser

This study investigates the association between IQ and risk for alcohol use disorder and the nature of this association.

## Introduction

The heritability of alcohol use disorder (AUD) is estimated to be approximately 50%,^1^ but most risk alleles identified to date each account for a very small proportion of the variance in liability to the disorder.^2,3,4^ Insights into the phenotypic expression of AUD risk may inform the development of psychosocial, behavioral, and pharmacologic interventions for the disorder. However, how genetic risk for AUD is expressed phenotypically remains not well understood, except for the role of genetic variation in alcohol-metabolizing enzymes^5^ and impulsivity.^6^

Measures of general intelligence (IQ) are substantially heritable, associated with multiple life outcomes,^7^ and may influence substance use disorder (SUD) risk. Results from twin studies show genetic overlap between measures of cognitive performance and AUD risk,^8^ and lower IQ has been observed in individuals with AUD—both before and after the development of alcohol-related problems.^9^ Furthermore, cognitive deficits are commonly found in individuals with AUD, with substantial recovery occurring during abstinence, suggesting an important role for environmental factors, including alcohol use itself.^10^ Even without AUD, moderate alcohol use is associated with negative cognitive effects.^11^ Nonetheless, it is not well understood how IQ is related to AUD risk. Additionally, although related to IQ, cognitive performance and educational attainment (EA) reflect distinct constructs whose associations with AUD may differ from those of IQ.

Here, we examined the association of these factors with AUD risk using several complementary approaches. First, using longitudinal data from a Swedish conscription cohort, cross-linked with population registers,^12^ we examined AUD risk as a function of IQ measured at approximately age 18 years while controlling for genetic and environmental confounds. We then used mendelian randomization (MR) analyses^13^ to evaluate potential causal effects of genetically predicted cognitive performance on AUD. Recognizing that the expression and consequences of cognitive traits varies across contexts,^14^ we used summary statistics from genome-wide association studies (GWAS) in US, UK, and Australian cohorts^3^ and a Finnish cohort.^15^ Finally, in an independent US sample, we evaluated associations between cognitive performance polygenic scores (PGS)—an aggregate measure of genetic liability—and AUD risk.

## Methods

 The Swedish Ethics Review Authority provided approval for use of pseudo-anonymized register data in the conscription cohort analyses. Swedish regulations do not require informed consent to be obtained for this type of research. The MR analyses did not involve individual-level data; only summary statistics were used. The PGS analyses were carried out under approval by the University of Pennsylvania and Yale University institutional review boards; all participants provided written informed consent. The eMethods in Supplement 1 contains further details on study methods. This study followed the Strengthening the Reporting of Observational Studies in Epidemiology (STROBE) reporting guidelines.

From a national cohort of males, the Swedish Military Conscription Register (coverage approximately 90%),^12^ we included individuals with IQ measures and no prior diagnosis of an SUD, which includes AUD (eFigure 1 in Supplement 1). IQ measures from a validated test battery^12^ were expressed on a stanine scale, with 5 corresponding to IQ = 100. We grouped IQ into low (1-3 points; ≥1 SD below the population mean), medium (4-6 points), and high (7-9 points; ≥1 SD above the population mean) groups to allow for clinical interpretation. AUD status was based on clinical diagnoses or alcohol-related death data extracted and cross-linked from national registers.^16^ Because registers capture different aspects of AUD, combining their data yields a more accurate model than relying on a single register.^17,18^ Data on comorbidities, siblings and parents, SUDs, birth year, EA, and housing were sourced from national registers (eTable 1 in Supplement 2). The epidemiological study is a Swedish national cohort. Males aged 18 years were included irrespective of race or ethnicity, and the population was predominantly of European descent. For the MR and PGS analyses, genetically inferred ancestry was used. This should not be considered a proxy for race or ethnicity.

### Statistical Analysis

We used Cox models to examine the association of IQ at conscription with lifetime AUD risk (eTable 2 in Supplement 2). Time to event was years from birth to first AUD diagnosis or AUD-related death, death from other causes, or end of follow-up. Birth years were used as strata to control for secular trends, and analyses were adjusted for diagnoses of internalizing conditions (depression or anxiety), attention-deficit/hyperactivity disorder (ADHD), and parental SUD. Although EA is often used as a proxy for socioeconomic status (SES), it correlates with IQ. Thus, we used a measure of household crowding during childhood as a proxy for SES in the adjusted analyses. Mediation analyses explored the extent to which the effect of IQ on AUD risk was mediated through EA, internalizing conditions, or ADHD, adjusted for parental SUD, childhood SES, and birth year (eTable 3 in Supplement 2).

#### Sensitivity Analysis

We performed several sensitivity analyses. First, to account for the potential that AUD was present but not diagnosed at conscription, we excluded the 5-year period after conscription before starting the follow-up, at which point 99.2% of individuals had not developed any SUD. Second, we compared AUD risk between siblings discordant for IQ group, controlling for birth year as 5-year strata, anxiety, depression, and ADHD diagnoses in each individual (eTable 4 in Supplement 2). Finally, we explored whether the effect of IQ on AUD risk differed across the IQ distribution using stanine points (point range, 1-9). Based on Akaike information criterion, a nonlinear model had a slightly better fit than a model using ranked categories. Using predictive margins, we visualized and compared the slope of AUD risk across the IQ distribution (eFigure 2 in Supplement 1 and eTable 5 in Supplement 2). Analyses were conducted in Stata, version 17 (StataCorp).

#### MR Analysis

We used MR analyses to examine the relationship between genetic liability for cognitive performance^19^ and both AUD^2^ and alcohol consumption^20^ (eFigure 3 in Supplement 1). Given prior evidence linking EA to AUD^21^ and the strong genetic correlation between cognitive performance and EA,^19,22^ we also conducted MR analyses using EA^23^ and the cognitive and noncognitive components of EA (hereafter referred to as *CogEA* and *NonCogEA*, respectively) as exposures.^22^ Because single-nucleotide variants (SNVs; previously referred to as *single-nucleotide polymorphisms* or *SNPs*) are fixed at conception, MR reduces residual confounding and limits reverse causation.^24,25,26^ Data sources for MR analyses are in eTable 6 in Supplement 2.

Instruments were genome-wide significant SNVs associated with the exposure, clumped at *R*^2^ = 0.001. Instrument strength was evaluated using *F* statistics (eTables 7-9 in Supplement 2), which suggested a low potential for weak instrument bias. We used inverse-variance–weighted MR (IVW), supplemented by MR-Egger, weighted median, mode-based, and MR-Lasso methods.^13^ For MR analyses with evidence of heterogeneity (Cochran *Q*, 2-sided *P* < .05), we used MR-Lasso to generate outlier-corrected estimates. Given that GWAS typically assess AUD and cognitive performance in adulthood, we used Steiger directionality tests to evaluate whether the proposed direction of effect (eg, cognitive performance → AUD) was supported (eTable 10 in Supplement 2).

We used single-variable MR to estimate effects of cognitive performance on potential confounders/mediators (ie, EA^22,23^ and psychiatric disorders^27,28,29^) and their effects on AUD (eTables 11 and 12 in Supplement 2).^30^ To assess whether cognitive performance has effects on AUD independent of other traits, we performed multivariable MR using genetic instruments for cognitive performance alongside EA, schizophrenia,^28^ depression,^29^ and ADHD^27^ (eFigure 4 in Supplement 1 and eTable 13 in Supplement 2).^30,31^ These models used multivariable extensions of IVW, MR-Egger, weighted median, and MR-Lasso. We evaluated horizontal pleiotropy and heterogeneity using the MR-Egger intercept and Cochran *Q* test, respectively.^30^ We conducted MR-based mediation models to examine whether effects of cognitive performance on AUD and alcohol consumption are mediated by EA or psychiatric conditions (eFigure 5 in Supplement 1 and eTable 14 in Supplement 2).

To evaluate whether genetic liability for cognitive performance is associated with individual-level AUD risk, we also generated cognitive performance PGS and tested associations with AUD in individuals of European-like genetic ancestry (EUR) from the US-based Yale-Penn cohort.^32,33^ PGS were generated using polygenic risk score continuous shrinkage (PRS-CS)^34^ and standardized. AUD diagnosis was based on participants’ responses to a semistructured assessment^35^ that included all 11 *DSM-5* AUD criteria. We used logistic regression to examine the association between cognitive performance PGS and AUD diagnosis, adjusting for age, sex, and the first 10 genetic principal components.

## Results

### Epidemiological Association of IQ and AUD Risk

**Figure 1.**
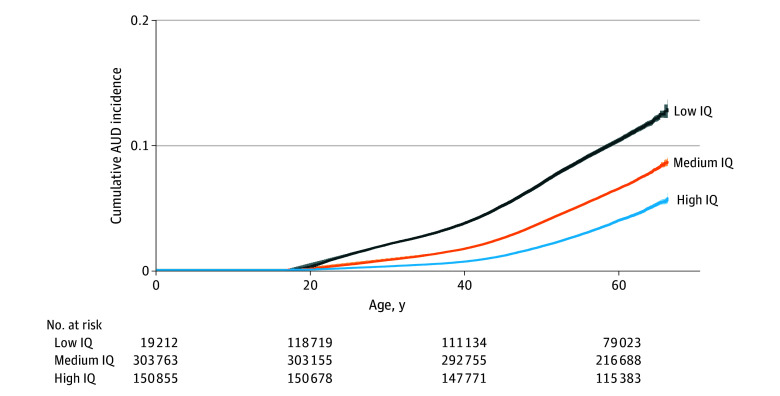
Inverse Association Between IQ Measured in Male Participants at Age 18 Years and Subsequent Alcohol Use Disorder (AUD) Risk Displayed is a crude cumulative incidence rate of AUD during follow-up across 3 IQ levels: low (≥1 SD below population mean), medium (within 1 SD of population mean), and high (≥1 SD above population mean). Shaded areas indicate 95% CIs.

Included in this study was a national cohort of 645 488 males, born between 1950 and 1962, from the Swedish Military Conscription Register, of whom 573 855 individuals were included in this analysis. All individuals were aged 18 years at IQ assessment, and mean (SD) follow-up time was 60.5 (7.9) years. Data on comorbidities, siblings and parents, SUDs, birth year, EA, and housing were sourced from national registers and included 191 928 individuals in 87 381 full-sibling clusters that ranged from 2 to 8 members, with most (n = 153 412) comprising 2 siblings. Descriptive statistics for the conscription cohort are shown in the Table. Crude cumulative incidence rates for AUD by IQ level (low, medium, high) are in Figure 1. There was a monotonic inverse association between IQ levels and lifetime prevalence of AUD.

**Table.** Characteristics of Swedish Males Who Completed Cognitive Testing at Study Inclusion, for the Overall Cohort, and Grouped by Low (1 ≥SD Below Population Mean), Medium (Within 1 SD of Population Mean), and High (≥1 SD Above Population Mean) Levels of IQ

Characteristic	No. (%) or mean (SD)				*P* value^a^
	Overall	IQ			
		Low	Medium	High	
Sample size	573 855	119 217 (20.8)	303 778 (52.9)	150 860 (26.3)	NA
Follow-up time, y	60.5 (7.9)	59.0 (9.7)	60.6 (7.7)	61.7 (6.4)	NA
Age at IQ testing, y	18.5 (0.9)	18.6 (1.3)	18.4 (0.84)	18.4 (0.77)	NA
Alcohol use disorder	37 333 (6.5)	11 876 (10.0)	19 434 (6.4)	6023 (4.0)	<.001
Parental SUD (including alcohol)	53 850 (9.4)	13 793 (11.6)	29 274 (9.6)	10 783 (7.2)	<.001
Household crowding	98 382 (17.1)	30 299 (25.4)	52 754 (17.4)	15 329 (10.2)	<.001
ADHD	3471 (0.6)	1268 (1.1)	1706 (0.6)	497 (0.3)	<.001
Internalizing conditions	53 417 (9.3)	15 508 (13.0)	27 544 (9.1)	10 366 (6.9)	<.001

Abbreviations: ADHD, attention-deficit/hyperactivity disorder; NA, not applicable; SUD, substance use disorder.

^a^ *P* values derived from Pearson χ^2^ test.

Low IQ was associated with a 64% higher lifetime AUD risk (crude HR, 1.64; 95% CI, 1.60-1.67) and a 43% higher risk (adjusted HR, 1.43; 95% CI, 1.40-1.47) after adjusting for parental SUD, internalizing conditions, ADHD, low SES, and birth year strata. Conversely, high IQ was associated with 40% reduced AUD risk (adjusted HR, 0.60; 95% CI, 0.59-0.62). Similar effects were found when follow-up started 5 years after conscription (adjusted HR, 1.40; 95% CI, 1.36-1.43) (eTable 2 in Supplement 2). IQ, internalizing conditions, and ADHD each had significant direct effects on AUD risk. In addition, mediation analysis indicated that a small but significant proportion (14%) of the effect of IQ on AUD risk was mediated through EA. In contrast, internalizing conditions and ADHD had negligible mediated effects (eTable 3 in Supplement 2). Sibling-pair analyses yielded similar results. Compared with their sibling with medium IQ, those with low IQ had nearly 40% increased risk for AUD (adjusted HR, 1.37; 95% CI, 1.22-1.55) after adjusting for internalizing conditions, ADHD, and birth year as 5-year strata (eTable 4 in Supplement 2). In the adjusted within-sibling comparison, the protective effect of high IQ was not significant. Finally, IQ as a continuous variable showed a nonlinear effect, with a steeper curve for lower than higher IQ, with a predicted effect on AUD risk of approximately 30% for the lowest IQ levels and only approximately 6.5% for higher IQ levels (eTable 5 in Supplement 2).

### MR Analyses

**Figure 2.**
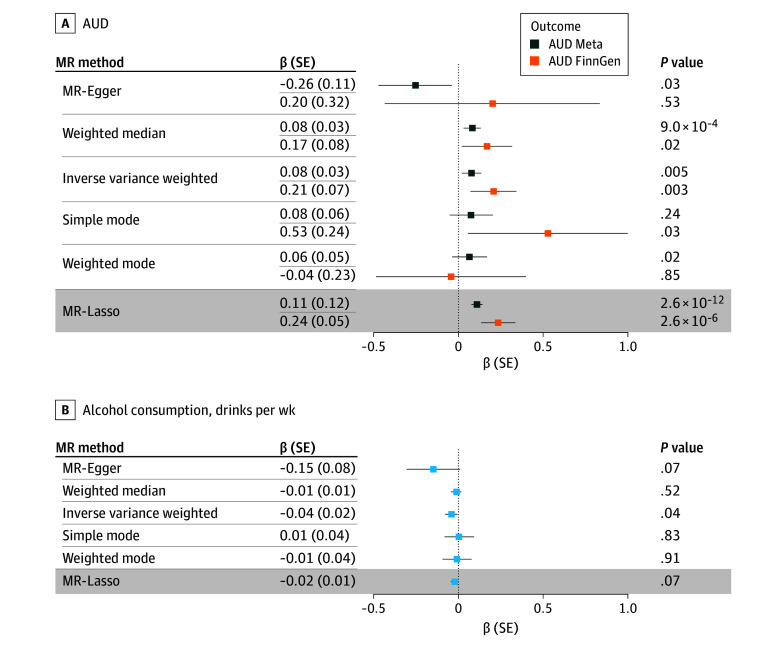
Mendelian Randomization (MR) Analysis of the Association Between Cognitive Performance and Alcohol Consumption With Alcohol Use Disorder (AUD) A, Results for AUD. B, Results for alcohol consumption. The MR-Lasso models were chosen as the primary model due to consistent evidence of pleiotropy. Additional models are reported for completeness. The dashed vertical line indicates a β of 0, with values crossing this line being nonsignificant. Shading indicates the results of the primary analysis.

MR analyses included summary statistics from GWAS of cognitive performance (n = 257 481) and AUD (total = 753 248; cases = 113 325) in individuals of European-like genetic ancestry (EUR), with FinnGen AUD GWAS as a replication sample (total = 500 348; cases = 20 597). Given consistent evidence of pleiotropy, we reported MR-Lasso as the primary MR method (Figure 2). Across all models, Steiger tests identified low risk of bias due to reverse causation. Lower genetic liability for cognitive performance was associated with increased AUD risk (β [SE], 0.11 [0.02]; *P* = 2.6 × 10^−12^), which was replicated in FinnGen.^15^ Lower EA^19^ was also associated with increased AUD risk (β [SE], 0.19 [0.01]; *P* = 1.0 × 10^−41^) and replicated in FinnGen. Both CogEA and NonCogEA had causal effects on increased AUD risk (CogEA: β [SE], 0.05 [0.01]; *P* = 2.56 × 10^−12^; NonCogEA: β [SE], 0.06 [0.01]; *P* = 4.65 × 10^−6^) that were replicated in FinnGen. In contrast to AUD, effects of cognitive performance, EA, CogEA, and NonCogEA on alcohol consumption were not significant (eTable 10 in Supplement 2).

Lower genetic liability for cognitive performance was associated with lower EA (β [SE], −0.45 [0.01]; *P* < 1.00 × 10^-300^) and increased risk of schizophrenia (β [SE], 0.38 [0.06]; *P* = 4.77 × 10^−11^) and ADHD (β [SE], 0.49 [0.05]; *P* = 7.3 × 10^−24^) (eTable 11 in Supplement 2). Effects on major depression were smaller but significant (β [SE], 0.08 [0.02]; *P* = 5.10 × 10^−5^). There were causal effects of all 3 psychiatric disorders on increased risk for AUD and greater alcohol consumption (eTable 12 in Supplement 2).

**Figure 3.**
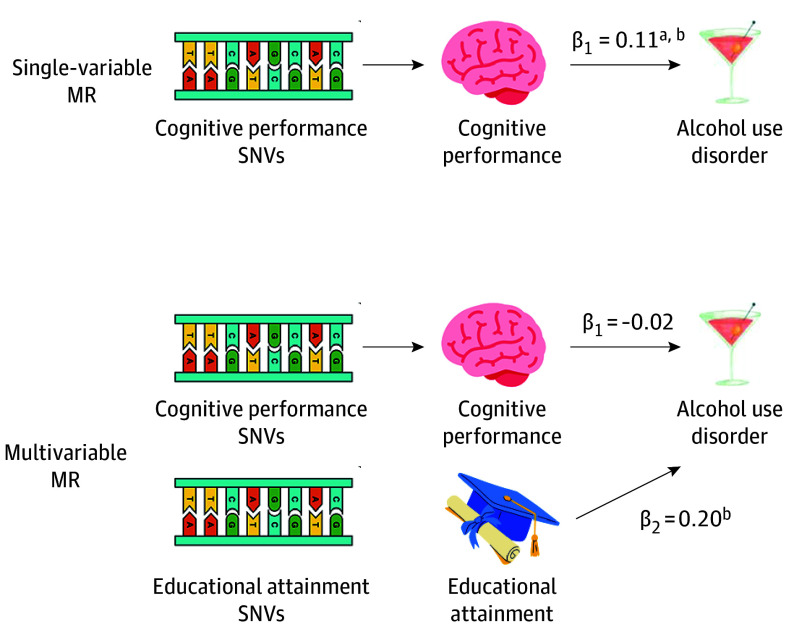
Multivariable Mendelian Randomization (MR) Results In single-variable MR, lower cognitive performance significantly predicted alcohol use disorder (AUD). In multivariable MR analysis, the direct effect of lower cognitive performance on alcohol use disorder was attenuated to null after accounting for the significant effect of educational attainment. This suggests a mediation of the cognitive performance effect through educational attainment in the main AUD Meta, a predominantly US- and UK-based analysis population. This mediation was not seen in corresponding analyses carried out in the Finnish FinnGen replication dataset, indicating that social context may shape how effects of low cognitive performance on AUD risk are mediated. SNV indicates single nucleotide variant. ^a^Indicates the total effect. ^b^*P* <.05.

Controlling for EA, lower genetic liability for cognitive performance had no direct effect on AUD (β [SE], −0.02 [0.02]; *P* = .22), but EA did (β [SE], 0.20 [0.02]; *P* = 2.46 × 10^−41^) (Figure 3). Analyses in FinnGen showed the opposite pattern, with the effect of cognitive performance larger after accounting for EA (β [SE], 0.56 [0.05]; *P* = 4.08 × 10^−29^) but showing no direct effect of EA (β [SE], 0.04 [0.05]; *P* = .40). Controlling for NonCogEA rendered the effect of cognitive performance on AUD nonsignificant (β [SE], 0.01 [0.02]; *P* = .61), which was replicated in FinnGen. In models that include EA and cognitive performance, only lower genetic liability for EA had effects on alcohol consumption (drinks per week: β [SE], 0.02 [0.01]; *P* = .03). When cognitive performance and NonCogEA were both included in the model, neither had direct effects on alcohol consumption (eTable 13 in Supplement 2). Formal mediation analyses were generally nonsignificant due to large SEs (eTable 14 in Supplement 2), but an estimated 55% of the effect of cognitive performance on AUD was mediated by EA (SE = 0.50; *P* = .27).

Effects of lower genetic liability for cognitive performance on AUD persisted in models with schizophrenia (β [SE], 0.11 [0.02]; *P* = 2.40 × 10^−10^) or ADHD (β [SE], 0.11 [0.02]; *P* = 2.99 × 10^−11^) but not depression (β [SE], −0.03 [0.02]; *P* = .10). However, in FinnGen, cognitive performance was significant after including depression (β [SE], 0.28 [0.06]; *P* = 1.27 × 10^−6^). Like the primary AUD results, direct effects of cognitive performance on alcohol consumption were significant after accounting for schizophrenia or ADHD but not depression. There was a significant indirect effect of cognitive performance through major depression on AUD (β [SE], 0.10 [0.04]; *P* = .01). Other indirect effects were nonsignificant.

### Associations With PGS

**Figure 4.**
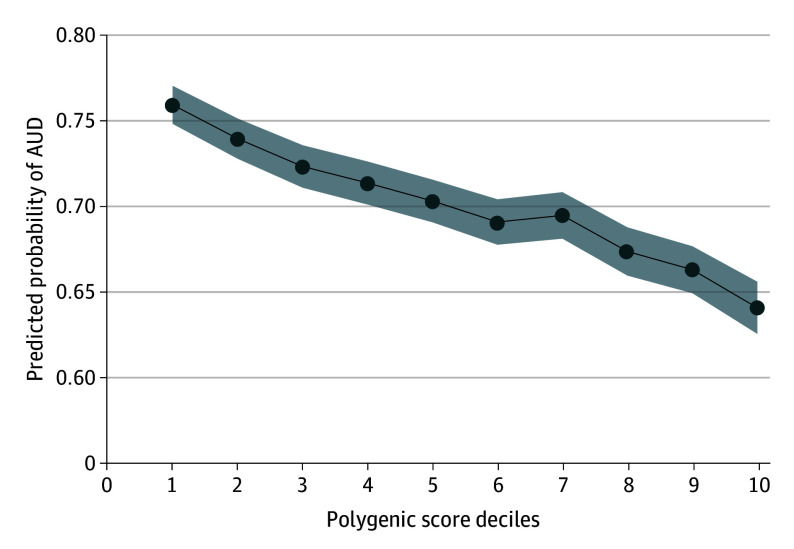
Polygenic Liability for Cognitive Performance and Prediction of Risk of Alcohol Use Disorder (AUD) Model predicted probabilities of alcohol use disorder in the US-based Yale-Penn sample are presented on the y-axis across deciles of the cognitive performance polygenic scores (x-axis). Grey shading represents error in the predicted probabilities.

To evaluate whether genetic liability for cognitive performance is associated with individual-level AUD risk, we also generated cognitive performance PGS and tested associations with AUD in individuals of EUR ancestry from the US-based Yale-Penn cohort (n = 5424). In that cohort, cognitive performance PGS were associated with 16.7% decreased odds of an AUD diagnosis (OR, 0.83; 95% CI, 0.78-0.89) (Figure 4). Compared with individuals in the lowest decile of cognitive performance PGS, those in the highest decile had 35% decreased odds of AUD (OR, 0.65; 95% CI, 0.48-0.88). Using decile 5 (ie, the approximate midpoint of the PGS distribution) as the reference category yielded similar results (eTable 15 in Supplement 2). There was no evidence for nonlinear PGS effects (eTable 15 in Supplement 2).

## Discussion

In a male Swedish national cohort linked with national health care and sociodemographic registers, IQ assessed at age 18 years was inversely associated with AUD risk, and the association remained robust in within-sibling comparisons that partially control for shared genetic and environmental factors. This effect was most pronounced at the lower end of the IQ distribution, with lower IQ conferring more risk than higher IQ conferring protection. MR analyses suggested a causal relationship between genetic liability for cognitive performance and AUD risk but identified context-dependent differences in the role of EA in explaining this association. Similarly, in a US-based cohort, individuals with the highest cognitive performance PGS had reduced odds of AUD compared with those with average PGS, but those with the lowest PGS did not have increased risk, contrasting with the Swedish findings. These results suggest that sociocultural factors like educational access and state support systems may shape how cognitive traits influence AUD risk.

AUD is moderately heritable (approximately 50%).^1^ However, the mechanisms by which genetic risk translates into the development of the disorder and how these mechanisms vary across environments are not well understood.^36^ Impulsive personality traits have received attention^6^ for their role in the development of SUDs, with support from genetic research. For example, in US college students, genetic liability for risk taking had effects on alcohol consumption through impulsivity.^37^ Our results identify cognitive traits as another key component of liability, with MR analyses consistent with a causal pathway from cognitive performance to AUD risk.

Importantly, SES may intersect with this pathway. Lower SES is associated with worse AUD-related health outcomes^38^ and greater AUD mortality.^39^ Although evidence linking SES to AUD incidence is weaker, it is still substantial.^40^ SES is commonly operationalized using EA,^41^ which is itself heritable and genetically correlated with cognitive performance (genetic correlation *r* approximately 0.65). Cognitive performance may confer resilience through enhanced problem-solving and adaptive coping during adversity,^7^ while EA reflects a broader constellation of genetically influenced traits, including cognitive performance, self-efficacy, personality, well-being, and behavioral regulation.^42^

In the male cohort from Sweden, a society with lower health and income inequality than the US,^43^ the effect of IQ on AUD remained even when controlling for an indicator of low SES. This suggests that the association between IQ and AUD risk cannot be fully explained by socioeconomic disadvantage. One interpretation of this is that genetic liability for IQ contributes to the observed associations among SES, EA, and AUD. This does not negate the influence of structural disadvantages or environmental adversity, but it does highlight the value of considering genetically influenced cognitive traits as a potential pathway linking SES and health.

MR analyses revealed context-dependent differences in causal pathways from genetic liability for cognitive performance to AUD risk. Using a meta-analysis of AUD derived from US, UK, and Australian cohorts, there was no direct effect of genetic liability for cognitive performance on AUD after controlling for EA. Instead, EA mediated 55% of the total effect of cognitive performance on AUD, although there was considerable imprecision in this estimate. Results using the Finnish AUD GWAS diverged, such that controlling for EA strengthened the effect of cognitive performance on AUD risk, in line with a suppressor effect. Rather than operating as a potential mediator along the risk pathway, in the Finnish cohort, EA buffered risks associated with lower genetic liability for cognitive performance. The findings suggest that causal pathways from cognitive performance to alcohol use outcomes are not universal and that structural conditions can magnify or mitigate the effects of genetic predispositions.

### Limitations

Our study has several limitations. IQ measures in the Swedish conscription cohort were based on comprehensive and validated testing, whereas the genetic analyses used results from GWAS that combined performance across a variety of cognitive assessments that varied in rigor.^19^ Additionally, because AUD classification in the Swedish conscription cohort was based on diagnoses in specialized care and on alcohol-related mortality, the most severe end of the AUD spectrum may have been overrepresented. Because we only had access to 3 levels of *International Statistical Classification of Diseases and Related Health Problems *(*ICD*) codes, we may have missed some alcohol-related medical diagnoses (eg, alcohol cardiomyopathy). Thus, we may have underestimated the prevalence of AUD in the Swedish male population. Swedish conscription data were only available for males, potentially limiting generalizability of the findings to females. However, PGS associations with AUD in the US-based Yale-Penn cohort, which comprises male and female participants, found similar results.

The genetic analyses also have limitations. First, although Steiger directionality tests consistently supported the hypothesized causal direction, reverse causation cannot be ruled out. However, results from the Swedish conscription cohort, which use IQ assessed at age 18 years before SUD onset and show persistent associations in within-sibling comparisons, offer additional support for the causal effects. Second, GWAS used in our analyses, including for AUD, are typically based on *ICD* codes, which may lack diagnostic specificity and introduce phenotypic heterogeneity. Third, the genetic analyses included only EUR participants because well-powered GWAS of IQ and other cognitive traits are not currently available in other groups. Insufficient representation of participants who are not of EUR ancestry hinders the discovery of potential population-specific variants and biomarkers for disease risk.^44^ Similarly, PGS developed using EUR GWAS results demonstrate poorer predictive performance in groups who are not of EUR ancestry.^45^ To address this, it is imperative to increase genetic diversity in genomic studies.^44^

## Conclusions

Taken together, findings of this study demonstrate that the association between cognitive traits and AUD was shaped by both genetic liability and the broader sociocultural environment in which that genetic liability is expressed. Across multiple methods and samples, cognitive performance and IQ were associated with AUD risk, but the nature of the association varied across contexts. We found that in settings with more equitable access to education and health care (ie, Sweden and Finland), EA may buffer, although not eliminate, the risk for AUD associated with lower cognitive performance/IQ. In more stratified social systems, by contrast, EA may amplify the vulnerability associated with lower cognitive liability by exacerbating disparities in access to health and opportunity. Our findings show that polygenic traits like cognitive performance, IQ, and EA did not exert uniform effects across populations. Thus, the interpretation and generalizability of genetic findings requires careful attention to the specific contexts in which the findings are observed. Efforts to mitigate AUD risk should account not only for individual differences in cognitive traits but also the structural conditions that shape how these differences manifest in health outcomes.

## Notes

Supplement 1.
eMethods.
**eFigure 1.** Flow Chart of Study Population Recruitment From the Swedish National Military Conscription Register **eFigure 2.** Effect of Different Levels of IQ Measured at Conscription on AUD Risk Later in Life **eFigure 3.** Directed Acyclic Graph (DAG) of the Mendelian Randomization Method **eFigure 4.** Multivariable MR (MVMR) DAG **eFigure 5.** MR Mediation With Difference Method
eReferences.

Supplement 2. **eTable 1.** List of Swedish National Registers and Variables Used **eTable 2.** Output Cox Models **eTable 3.** Mediation Analysis—Proportion of the Effect of IQ on AUD Risk Mediated Through Education, ADHD, Stress/Anxiety Respectively **eTable 4.** Sibling Comparison **eTable 5.** Nonlinear Models Using IQ Assessed at Conscription used as Continuous Variable **eTable 6.** Phenotypes **eTable 7.** Cognitive Performance Instruments **eTable 8.** Educational Attainment Instruments **eTable 9.** Cognitive and Noncognitive Components of Educational Attainment Instruments **eTable 10.** Single Variable Mendelian Randomization Results **eTable 11.** Single Variable Mendelian Randomization Results for Cognitive Performance on Mediators **eTable 12.** Single Variable Mendelian Randomization Results of Psychiatric Traits on Alcohol-Related Traits **eTable 13.** Multivariable Mendelian Randomization Results **eTable 14.** Mendelian Randomization Mediation Model Results **eTable 15.** Associations of Polygenic Scores With Alcohol Use Disorder Yale-Penn

Supplement 3.
Data Sharing Statement.
