# Supplementary material for: Measures of General Intelligence and Risk for Alcohol Use Disorder
Source: JAMA Psychiatry. 2025 Oct 1;82(12):1195–202. doi: 10.1001/jamapsychiatry.2025.2689 (PMC12489797; doi:10.1001/jamapsychiatry.2025.2689)
Supplement: Supplement 1. — eMethods. eFigure 1. Flow Chart of Study Population Recruitment From the Swedish National Military Conscription Register eFigure 2. Effect of Different Levels of IQ Measured at Conscription on AUD Risk Later in Life eFigure 3. Directed Acyclic Graph (DAG) of the Mendelian Randomization Method eFigure 4. Multivariable MR (MVMR) DAG eFigure 5. MR Mediation With Difference Method eReferences. [file jamapsychiatry-e252689-s001.pdf]

Supplementary Online Content

Capusan AJ, Davis C, Thern E, et al. Measures of general intelligence and risk for alcohol use disorder. *JAMA Psychiatry*. Published online October 1, 2025. doi:10.1001/jamapsychiatry.2025.2689

eMethods.

eFigure 1. Flow Chart of Study Population Recruitment From the Swedish National Military Conscription Register

eFigure 2. Effect of Different Levels of IQ Measured at Conscription on AUD Risk Later in Life

eFigure 3. Directed Acyclic Graph (DAG) of the Mendelian Randomization Method

eFigure 4. Multivariable MR (MVMR) DAG

eFigure 5. MR Mediation With Difference Method

eReferences.

This supplementary material has been provided by the authors to give readers additional information about their work.

© 2025 Capusan AJ et al. *JAMA Psychiatry*.

eMethods

1 CONSCRIPTION REGISTER DATA

1.1. Participants

We used a national cohort of 645,488 males, born from 1950-1962, obtained from the Swedish Military Conscription Register (SMCR). Participants included in the analyses were those for whom IQ measures were available, and who did not have a substance use disorder (SUD, including alcohol use disorder (AUD)) diagnosis before or at conscription (n=573,855). Conscription was mandatory for men in Sweden until 2010. The coverage is therefore excellent, or ~90% among men born 1951 to 1988[1]. IQ at conscription was assessed at around age 18, with a validated test battery covering logical, verbal, spatial, and theoretical/technical aspects of reasoning[1,2]. Test results are expressed on a 9-point stanine scale, where 1 is lowest and 9 highest, and where 5 corresponds to an IQ of 100. We grouped IQ into three levels: low (1-3 points; appr. ≥1 standard deviation [SD] below the population mean), medium (4-6 points) and high (7-9 points; appr. ≥1 SD above the population mean). A flow chart on inclusion of participants from the Swedish National Military Conscription Register is shown in **Figure S1.**

Data on AUD status were extracted from national registers, cross-linked using the Swedish personal identity number[3]. Because different national registers capture different aspects of AUD[4], using a single register could underestimate the burden of disease, and a combination of different information sources renders a more accurate AUD diagnosis[5,6]. Subjects were classified as having AUD if they had an alcohol-related diagnosis according to the International Classification of Disease (ICD) 8, 9 or 10 (see **Table S1**) in the Swedish National Patient Register (covering both inpatient and specialized outpatient care), or had an alcohol-related cause of death recorded in the National Cause of Death Register. Data on comorbidities, parental SUD, age, education (EA), and housing were also sourced from relevant national registers (**Table S1**). Register data were irreversibly anonymized before delivery to the investigators to protect the integrity of the participants. The study was approved by the Swedish Ethical Review Authority (Dnr 2019–02161).

© 2025 Capusan AJ et al. *JAMA Psychiatry*.

1.2 Statistical analysis

We used Cox models to investigate the association of IQ at conscription with lifetime AUD risk **(Figure S2)**. Time to event was defined as years from birth to first AUD-related event (AUD diagnosis or AUD-related death), death from other diagnoses, or end of follow-up (31 December 2019; when the men were 57-69 years old). We used birthyears as strata to account for secular trends such as changes in health care access over time, and adjusted for diagnoses of internalizing conditions, defined as depression or anxiety diagnoses; for diagnosis of ADHD; and for parental SUD.

Because EA, often used as a proxy for socioeconomic status (SES), correlates with IQ, we used household crowding during childhood as a proxy for low SES in the adjusted analysis. Household crowding is defined by Statistics Sweden as living more than two individuals per room, excluding the kitchen[7].

Using mediation analyses we explored to what extent the effect of IQ on AUD risk was mediated through EA (lowest, vs. secondary and post-secondary), internalizing conditions (anxiety, depression) and ADHD. As above, mediation analyses were adjusted for parental SUD, and household crowding as a proxy for low SES during childhood and birthyear.

In the sibling comparison analysis, we used family ID and birthyear grouped every five-year as strata. We also tested the use of a cluster robust standard error, to account for potential within cluster correlations. However, this did not change estimates significantly and thus was not included in the final analysis. The results presented for the sibling analysis are stratified by family cluster and birthyear (five-year strata), including anxiety, depression and ADHD in the individual as covariates. Statistical analysis was performed in STATA (version 17).

© 2025 Capusan AJ et al. *JAMA Psychiatry*.

2 Mendelian Randomization analyses

2.1 Assumptions of Mendelian randomization (MR)

MR relies on three core assumptions for causal inference to be valid (**Figure S3**). First, SNPs must be associated with the exposure. This is ensured by using genome-wide significant SNPs from large-scale GWAS ($p < 5x10^{-8}$). To avoid weak instrument bias, we also examined instrument strength using the F-statistic, where instruments with $F < 10$ are generally considered weak and may bias estimates. Second, genetic instruments should affect the outcome only through their effect on the exposure, not via alternative pathways (e.g., horizontal pleiotropy). To assess and correct for potential pleiotropy, we used multiple complementary MR estimators: (1) MR-Egger, which tests for directional pleiotropy via the intercept term; (2) weighted median and mode-based MR, which yield valid estimates even when some instruments are invalid; and (3) MR-Lasso, which performs SNP selection and penalization to exclude invalid instruments. Finally, instruments must be independent of confounders of the exposure-outcome relationship[8-11]. In practice, this is the most difficult assumption to verify, as not all sources of confounding can be accounted for. However, to reduce the risk of violating this assumption, we used GWAS summary statistics that were adjusted for genetic ancestry principal components, which reduces bias from population stratification. We also interpret our findings considering this limitation.

2.2 Data sources

We obtained publicly available summary statistics from GWAS of cognitive performance, AUD, alcohol consumption (i.e., drinks per week), EA, and psychiatric conditions for use in the MR analyses (**Table S6**). All GWAS were conducted in populations of European-like genetic ancestry, with participant data obtained under approved ethical guidelines from institutional review boards with informed consent. MR analyses followed the STROBE-MR guidelines[12] (**Supplementary Checklist**).

2.2.1 Cognitive performance

We used summary statistics evaluating cognitive performance [N=257841[13]] to construct our genetic instrument. Lee et al. meta-analyzed UK Biobank fluid intelligence verbal-numerical

© 2025 Capusan AJ et al. *JAMA Psychiatry*.

reasoning test data and the Cognitive Genomics Consortium (COGENT) neuropsychological test data[13]. The UK Biobank fluid intelligence verbal-numerical reasoning score contained 13 logic and reasoning questions designed to measure fluid intelligence (data field 20016 for in-person assessments and data field 20191 for the online follow-up). UK Biobank participants had a two-minute time limit for the test and took the assessment up to four times[13]. Lee et al. took the mean of the standardized scores from 222543 UKB participants and then standardized this mean[13]. The COGENT data included 35 component studies (N=35298) and used a phenotype constructed from the unrotated principal component for the performance on 3+ neuropsychological tests or 2+ intelligence quotient (IQ) subscales [13]. These data were also standardized[13].

2.2.2 AUD and alcohol consumption outcomes

Our primary AUD outcome was from the GWAS meta-analysis of the Million Veteran Program [MVP], Psychiatric Genomics Consortium [PGC], QIMR, iPSYCH, and Yale-Penn cohorts [113,325 cases; 639,923 controls;[14]]. We also included an additional, independent AUD outcome using the latest release of the FinnGen study (R12) [20,597 cases; 479,751 controls; [15]]. The FinnGen AUD definition is based upon the International Classification of Disease (ICD) 8, 9, or 10 diagnoses for harmful alcohol use or alcohol dependence and ICD causes of death related to harmful alcohol use or alcohol dependence[15]. We also assessed the impact of cognitive performance on alcohol consumption. For this outcome, we used GWAS summary statistics from Saunders et al. (2022)[16] for drinks per week (n=666,978).

2.2.3 Educational attainment and psychiatric conditions

For Educational Attainment (EA), we obtained GWAS summary statistics for EA from the Social Science Genetic Association Consortium (SSGAC) analysis of years of schooling excluding 23andMe [N=765283; [17]]. The cognitive and non-cognitive components of EA were obtained from GWAS-by-subtraction analyses subtracting the effect of cognitive performance from EA GWAS[18]. We obtained schizophrenia (SCZ) summary statistics from the PGC meta-analysis of 90 cohorts [53,386 cases; 77,258 controls;[19]]. SCZ cases were based on clinical evaluation, semi-structured interviews, and medical records in accordance with DSM-IV or ICD-10 criteria. For

© 2025 Capusan AJ et al. *JAMA Psychiatry*.

depression, we used GWAS summary statistics from a meta-analysis of depression among participants from the UK Biobank, PGC, MVP, iPSYCH, and FinnGen [295,577 cases; 764,956 controls;[20]]. In the UK Biobank, cases of depression were defined as either 1) an answer of “Yes” to the question: “Have you ever seen a general practitioner/psychiatrist for nerves, anxiety, tension, or depression,” or 2) if participants met criteria for and were diagnosed with any of these depressive mood disorders as defined by the ICD codes: F32 – Single Episode Depression, F33 – Recurrent Depression, F34 – Persistent mood disorders, F38 – Other mood disorders, and F39 – Unspecified mood disorders. The PGC MDD data comprised 7 cohorts where cases were classified as having MDD through self-report, clinician interviews, and/or medical record diagnoses based on standard criteria[21]. Depression in MVP and iPSYCH was based on the presence of ICD-9 and ICD-10 codes in the electronic health records. Finally, the ADHD GWAS was from a meta-analysis of the iPSYCH, deCODE, and 10 PGC cohorts [38,691 cases; 186,843 controls [22]]. In iPSYCH, ADHD cases were identified using ICD-10 codes (F90.0, F90.1, F98.8). In deCODE, cases were either individuals with a clinical diagnosis of ADHD based on ICD-10 criteria or individuals prescribed medication specific for ADHD symptoms (ATC-NA06BA).

2.3 MR statistical analysis

2.3.1 Sensitivity analyses

Single variable MR with educational attainment: Given the previously identified role of EA in AUD [23] and strong genetic correlations between cognitive performance and EA [13,18], we performed single-variable MR analyses using exposures reflecting EA, as well as the cognitive and non-cognitive contributions to EA[18], in association with AUD and alcohol consumption outcomes.

Test of directionality: To assess whether the hypothesized direction of causality (i.e., from cognitive performance to AUD) was consistent with the data, we conducted Steiger directionality tests for the single variable MR models. The Steiger test compares the proportion of the variance explained by the genetic instruments for the exposure and the outcome. If the instruments explain more variance in the outcome than the exposure, it suggests possible reverse causation.

© 2025 Capusan AJ et al. *JAMA Psychiatry*.

Multivariable MR: The genetic architecture of cognitive performance is genetically correlated with psychiatric disorders[13], suggesting that the cognitive performance SNP instruments might affect AUD and through psychiatric pathways[24,25]. MVMR, an extension of single-variable MR, simultaneously examines relationships between multiple related exposures and an outcome[24,26]. MVMR incorporates SNPs across multiple exposures, enabling us to identify each exposure's independent (i.e., direct) effect (**Figure S4**)[24,26]. Thus, these models clarify the unique influence of cognitive performance on AUD after accounting for potential confounds. We conducted MVMR models for cognitive performance with education and with psychiatric disorders using multivariable extensions of IVW, MR Egger, weighted median, and MR-Lasso. We also conducted multivariable MR Egger and Cochran's Q test to assess for pleiotropy and heterogeneity in the instruments' effects[13,24].

2.3.2 MR Mediation analyses

We conducted MR mediation analyses to quantify how much of the effect of cognitive performance on AUD is mediated by EA, the non-cognitive components of EA, and psychiatric disorders. After running single and multivariable MR for each exposure and mediator pair, we used the difference method to estimate the indirect effect and proportion mediated[24] **(Figure S5)**. This method involves first estimating the total effect of the exposure on the outcome using single-variable MR and then calculating the direct effect using MVMR, where the effect of the exposure is conditioned on the potential mediator (e.g., EA or psychiatric disorders). The difference in the effect of the primary exposure across the two sets of analyses represents the indirect effect, or the effect of cognitive performance on AUD that is mediated by EA or psychiatric disorders. The proportion of the effect mediated is the indirect effect divided by the total effect which is obtained using single variable MR[24].

© 2025 Capusan AJ et al. *JAMA Psychiatry*.

2.4 Supplementary Checklist: STROBE-MR Reporting Guidelines

1. TITLE and ABSTRACT
Indicate Mendelian randomization as the study's design in the title and/or the abstract.
MR analyses are discussed in the abstract.

INTRODUCTION
2. Background
Explain the scientific background and rationale for the reported study. Is causality between exposure and outcome plausible? Justify why MR is a helpful method to address the study question.
Addressed in the Introduction and Methods.

3. Objectives
State specific objectives clearly, including pre-specified causal hypotheses (if any).
Addressed in the Introduction and Methods.

METHODS
4. Study design and data sources
Present key elements of study design early in the paper. Consider including a table listing sources of data for all phases of the study. For each data source contributing to the analysis, describe the following:
a) Describe the study design and the underlying population from which it was drawn. Describe also the setting, locations, and relevant dates, including periods of recruitment, exposure, follow-up, and data collection, if available.
b) Give the eligibility criteria, and the sources and methods of selection of participants.
c) Explain how the analyzed sample size was arrived at.
d) Describe measurement, quality and selection of genetic variants.
e) For each exposure, outcome and other relevant variables, describe methods of assessment and, in the case of diseases, the diagnostic criteria used.
f) Provide details of ethics committee approval and participant informed consent, if relevant.
Addressed in the Methods and Supplementary Methods.

5. Assumptions
Explicitly state assumptions for the main analysis (e.g. relevance, exclusion, independence, homogeneity) as well assumptions for any additional or sensitivity analysis.
Addressed in the Supplementary Methods.

6. Statistical methods: main analysis
Describe statistical methods and statistics used.
a) Describe how quantitative variables were handled in the analyses (i.e., scale, units, model).
b) Describe the process for identifying genetic variants and weights to be included in the analyses (i.e, independence and model). Consider a flow diagram.
c) Describe the MR estimator, e.g. two-stage least squares, Wald ratio, and related statistics.

© 2025 Capusan AJ et al. *JAMA Psychiatry*.

Detail the included covariates and, in case of two-sample MR, whether the same covariate set was used for adjustment in the two samples.
d) Explain how missing data were addressed.
e) If applicable, say how multiple testing was dealt with.
Addressed in the Methods and Supplementary Methods

7. Assessment of assumptions
Describe any methods used to assess the assumptions or justify their validity.
Addressed in the Methods and Supplementary Methods

8. Sensitivity analyses
Describe any sensitivity analyses or additional analyses performed.
Addressed in the Methods and Supplementary Methods

9. Software and pre-registration
a) Name statistical software and package(s), including version and settings used.
Addressed in the Methods.

b) State whether the study protocol and details were pre-registered (as well as when and where).
Addressed in the Methods.

RESULTS
10. Descriptive data
a) Report the numbers of individuals at each stage of included studies and reasons for exclusion. Consider use of a flow-diagram.
b) Report summary statistics for phenotypic exposure(s), outcome(s) and other relevant variables (e.g. means, standard deviations, proportions).
c) If the data sources include meta-analyses of previous studies, provide the number of studies, their reported ancestry, if available, and assessments of heterogeneity across these studies. Consider using a supplementary table for each data source.
d) For two-sample Mendelian randomization:
i. Provide information on the similarity of the genetic variant-exposure associations between the exposure and outcome samples.
ii. Provide information on extent of sample overlap between the exposure and outcome data sources.
Addressed in the Methods, Results, and Supplementary Tables.

11. Main results
a) Report the associations between genetic variant and exposure, and between genetic variant and outcome, preferably on an interpretable scale (e.g. comparing 25th and 75th percentile of allele count or genetic risk score, if individual-level data available).

© 2025 Capusan AJ et al. *JAMA Psychiatry*.

b) Report causal effect estimate between exposure and outcome, and the measures of uncertainty from the MR analysis. Use an intuitive scale, such as odds ratio, or relative risk, per standard deviation difference.
c) If relevant, consider translating estimates of relative risk into absolute risk for a meaningful time-period.
d) Consider any plots to visualize results (e.g. forest plot, scatterplot of associations between genetic variants and outcome versus between genetic variants and exposure).
Addressed in the Results and Supplementary Tables.

12. Assessment of assumptions
a) Assess the validity of the assumptions.
b) Report any additional statistics (e.g., assessments of heterogeneity, such as I2, Q statistic).
Addressed in the Results, Supplementary Tables, and Discussion.

13. Sensitivity and additional analyses
a) *Use sensitivity analyses to assess the robustness of the main results to violations of the assumptions.*
b) Report results from other sensitivity analyses (e.g., replication study with different dataset, analyses of subgroups, validation of instrument(s), simulations, etc.).
c) Report any assessment of direction of causality (e.g., bidirectional MR).
d) When relevant, report and compare with estimates from non-MR analyses.
e) Consider any additional plots to visualize results (e.g., leave-one-out analyses).
Addressed in the Results and Supplementary Tables.

DISCUSSION
14. Key results
Summarize key results with reference to study objectives.
Addressed in the Discussion.

15. Limitations
Discuss limitations of the study, taking into account the validity of the MR assumptions, other sources of potential bias, and imprecision. Discuss both direction and magnitude of any potential bias, and any efforts to address them.
Addressed in the Discussion.

16. Interpretation
a) Give a cautious overall interpretation of results considering objectives and limitations. Compare with results from other relevant studies.
b) Discuss underlying biological mechanisms that could be modelled by using the genetic variants to assess the relationship between the exposure and the outcome.
c) Discuss whether the results have clinical or policy relevance, and whether interventions could have the same size effect.
Addressed in the Discussion.

© 2025 Capusan AJ et al. *JAMA Psychiatry*.

17. Generalizability
Discuss the generalizability of the study results (a) to other populations (i.e. external validity), (b) across other exposure periods/timings, and (c) across other levels of exposure.
Addressed in the Discussion.

OTHER INFORMATION
18. Funding
Give the source of funding and the role of the funders for the present study and, if applicable, for the original study or studies on which the present article is based.
Addressed in the Funding.

19. Data and data sharing
Present data used to perform all analyses or report where and how the data can be accessed. State whether statistical code is publicly accessible and if so, where.
Addressed in the Methods.

20. Conflicts of Interest
All authors should declare all potential conflicts of interest.
Addressed in the Conflicts of interest.

© 2025 Capusan AJ et al. *JAMA Psychiatry*.

eFigure 1. Flow chart of study population recruitment from the Swedish National Military Conscription Register.

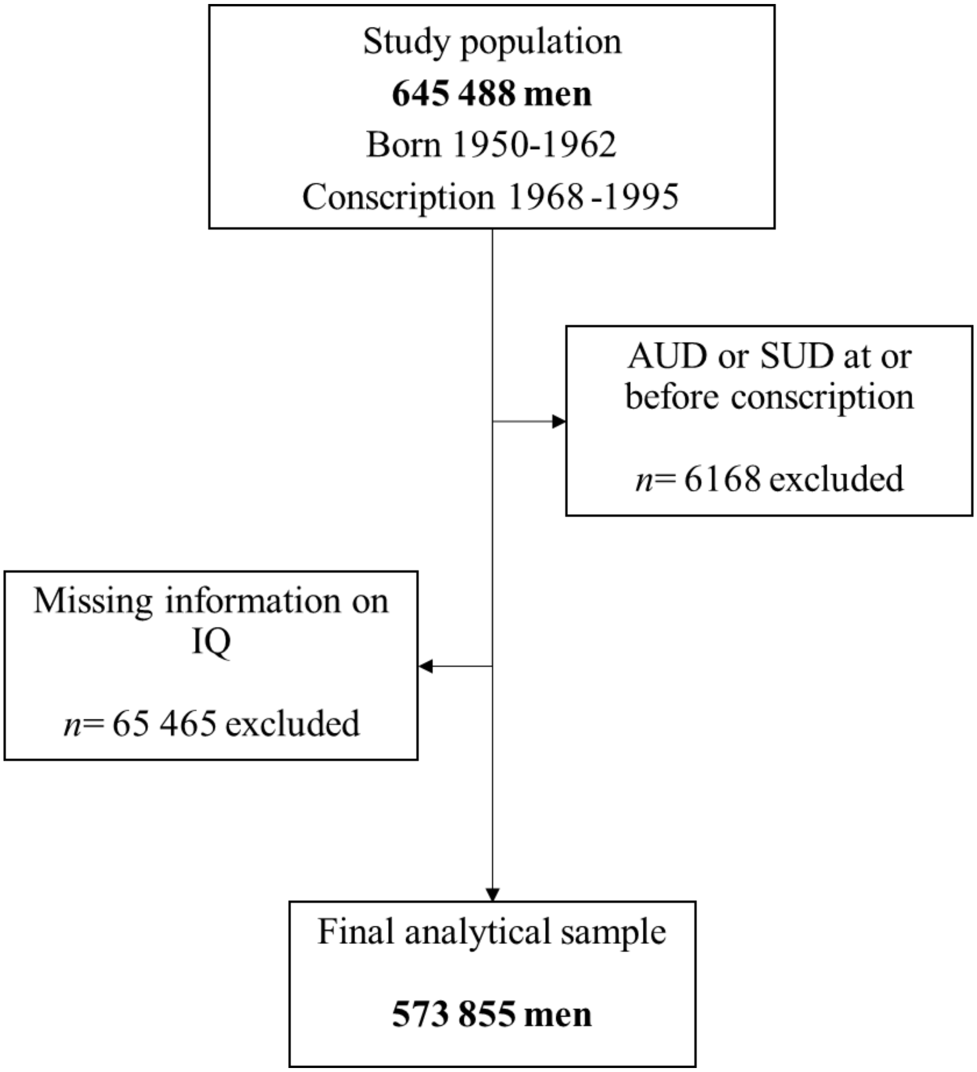

© 2025 Capusan AJ et al. *JAMA Psychiatry*.

eFigure 2 Effect of different levels of IQ measured at conscription on AUD risk later in life

Predictive margins calculated for the full sample (n= 573 855) for the AUD risk per IQ measures assessed at conscription (age 18). IQ is expressed in stanine points (1-9) used as a continuous variable

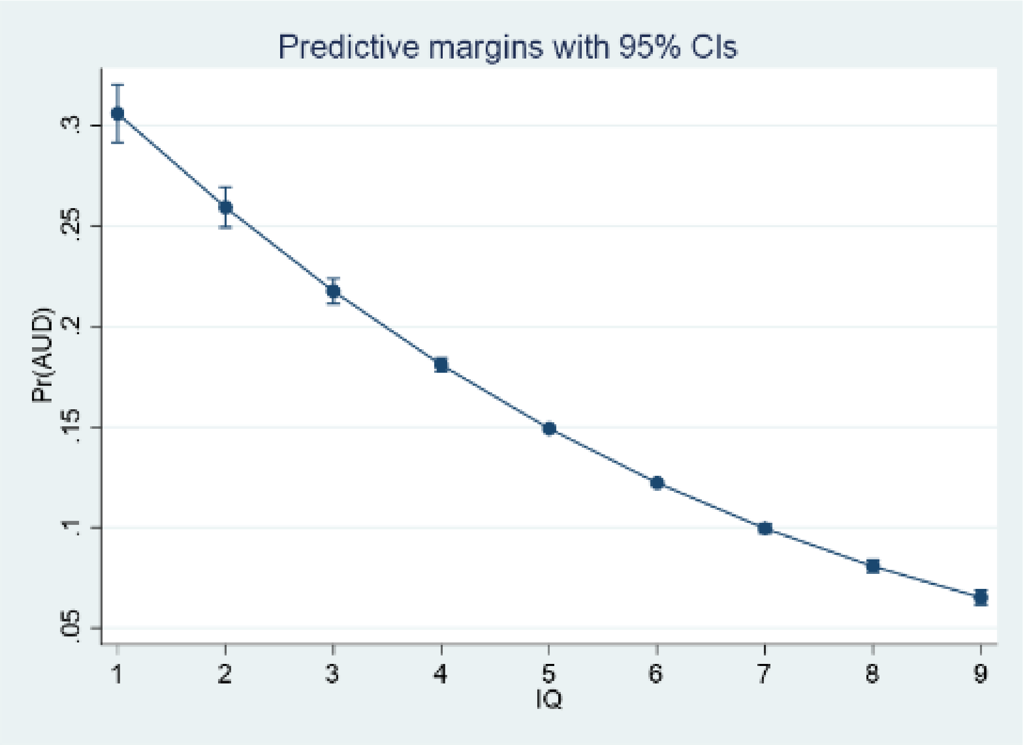

IQ level	Predictive margin	95%CI
1	0.31	(0.29, 0.32)
2	0.26	(0.25, 0.27)
3	0.22	(0.21, 0.22)
4	0.18	(0.18, 0.18)
5	0.15	(0.15, 0.15)
6	0.12	(0.12, 0.12)
7	0.10	(0.10, 0.11)
8	0.08	(0.08, 0.08)
9	0.07	(0.06, 0.07)

AUD: Alcohol use disorder
IQ: expressed in stanine points 1-9, with 5 corresponding an IQ level of around 100
CI: confidence interval

eFigure 3. Directed acyclic graph (DAG) of the Mendelian randomization method. DAG is adapted from Sanderson et al [25]. Mendelian Randomization (MR) is based upon on three

© 2025 Capusan AJ et al. *JAMA Psychiatry*.

essential assumptions: (1) Relevance Assumption (IV1): The genetic variants selected as instruments (genome-wide variants) must be strongly associated with the exposure of interest. This ensures the instrument's relevance for investigating causal effects. (2) Independence Assumption (IV2): The genetic variants must not be influenced by confounders—whether measured or unmeasured—that could also affect the outcome (Y). (3) Exclusion Restriction Assumption (IV3): The genetic variants must influence the outcome (Y) solely through their effect on the exposure (X), with no alternative pathways, including horizontal pleiotropy. These assumptions are crucial for ensuring the validity of MR analyses. Dotted lines in the accompanying diagram represent assumed non-existent relationships, highlighting the independence and exclusion restrictions central to the method.

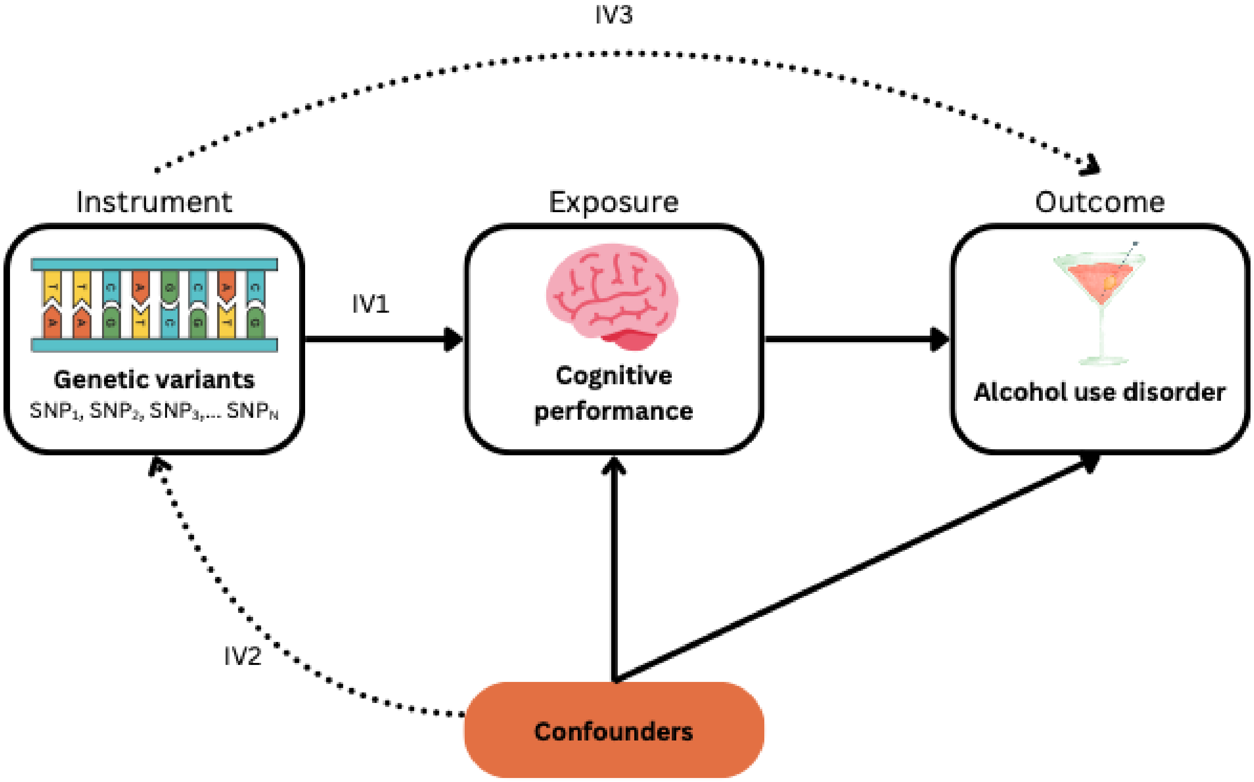

© 2025 Capusan AJ et al. *JAMA Psychiatry*.

eFigure 4. Multivariable MR (MVMR) DAG. MVMR DAG is adapted from Sanderson et al [25]. MVMR extends conventional single-variable MR methods (one exposure and one outcome) to estimate the total, indirect, and direct effects of multiple exposures on the outcome of interest. In this study, we use MVMR models that use genetic instruments (i.e., common single nucleotide polymorphisms) associated with both cognitive performance and possible educational and psychiatric mediators/confounders (Exposure 2 in the figure) to estimate the direct effect of cognitive performance on alcohol use disorder.

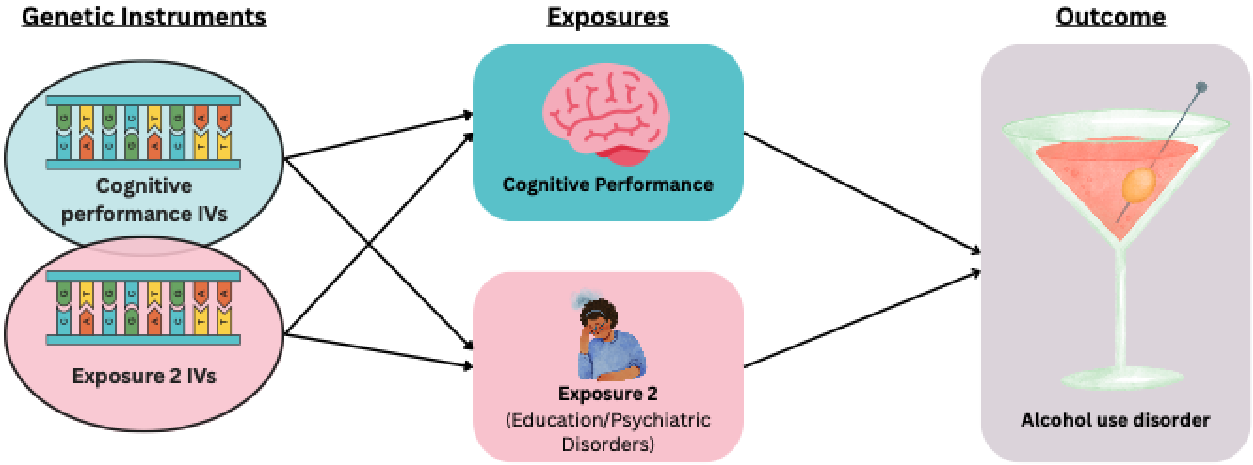

© 2025 Capusan AJ et al. *JAMA Psychiatry*.

eFigure 5. MR mediation with difference method. Adapted from Sanderson et al.[24], the figure outlines the directed acyclic graphs for single-variable (top) and multivariable MR (bottom) analyses assessing the impact of cognitive performance and educational attainment on alcohol use disorder (AUD) and how estimates from the two sets of analyses are used to examine potential mediation using the difference method. The total effect of cognitive performance on AUD is provided by the single-variable MR estimate (β_1*). The direct effect of cognitive performance on AUD, accounting for educational attainment, is the MVMR estimate (β_1). Similarly, the direct effect of educational attainment on AUD is its MVMR estimate (β_2). The indirect effect, which is the effect of cognitive performance on AUD that operates through educational attainment (shown by the blue dashed arrows), is calculated as the difference between the total and direct effects ($\beta_1* - \beta_1$). The proportion of the effect of cognitive performance on AUD that is mediated by educational attainment is calculated by dividing the indirect effect by the total effect.

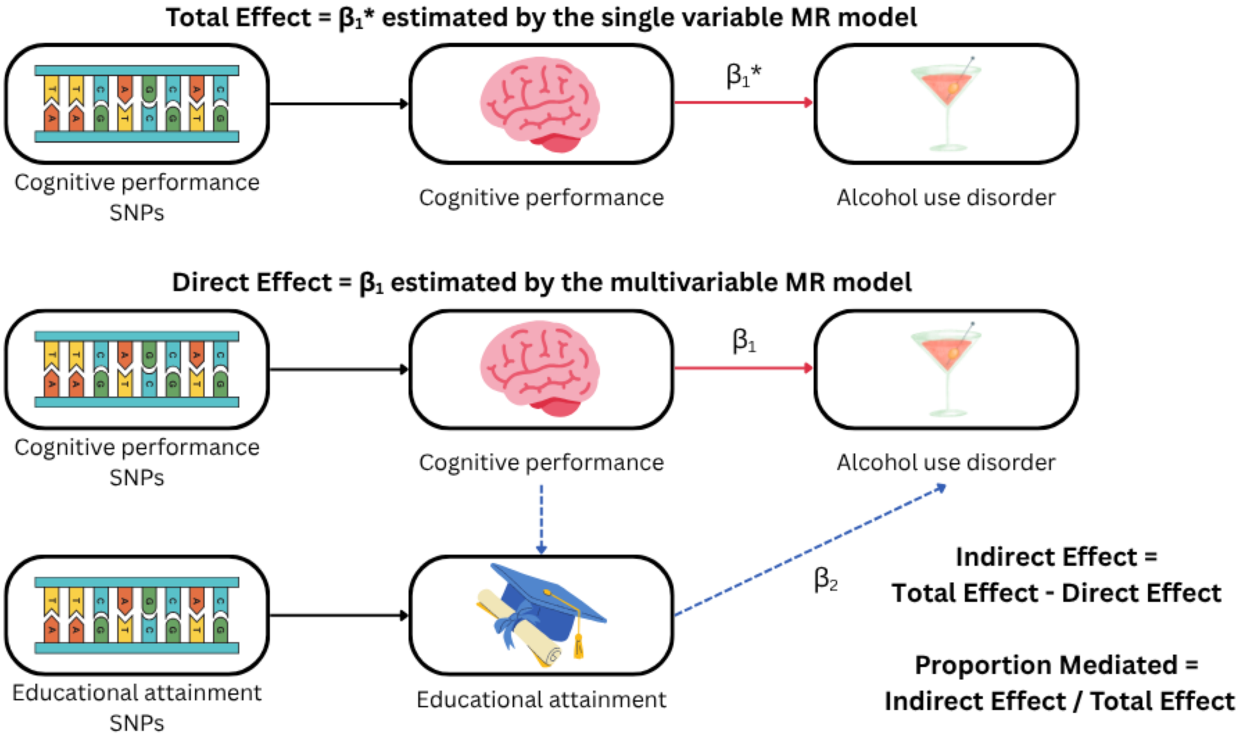

© 2025 Capusan AJ et al. *JAMA Psychiatry*.

eReferences.

1. Ludvigsson JF, Berglind D, Sundquist K, Sundström J, Tynelius P, Neovius M. The Swedish military conscription register: opportunities for its use in medical research. *Eur J Epidemiol*. Jul 2022;37(7):767-777. doi:10.1007/s10654-022-00887-0
2. Carlstedt B, Mårdberg B. Construct validity of the Swedish Enlistment Battery. *Scand J Psychol*. 1993;34(4):353-362. doi:https://doi.org/10.1111/j.1467-9450.1993.tb01131.x
3. Ludvigsson JF, Almqvist C, Bonamy AK, et al. Registers of the Swedish total population and their use in medical research. *Eur J Epidemiol*. Feb 2016;31(2):125-36. doi:10.1007/s10654-016-0117-y
4. Lundin A, Danielsson AK, Dalman C, Hollander AC. Indications of alcohol or drug use disorders in five different national registers in Sweden: a cross-sectional population-based study. *BMJ Open*. Sep 4 2023;13(9):e070744. doi:10.1136/bmjopen-2022-070744
5. Bergman D, Hagström H, Capusan AJ, et al. Incidence of ICD-based diagnoses of alcohol-related disorders and diseases from Swedish nationwide registers and suggestions for coding. *Clinical Epidemiology*. 2020:1433-1442.
6. Capusan AJ, Gustafsson PA, Kuja-Halkola R, Igelström K, Mayo LM, Heilig M. Re-examining the link between childhood maltreatment and substance use disorder: a prospective, genetically informative study. *Mol Psychiatry*. Apr 6 2021;26(7):3201-3209. doi:10.1038/s41380-021-01071-8
7. Statistics Sweden. *Housing Census 1960: Dwellings and households in the counties and in the whole country*. 1963.
8. Davey Smith G, Hemani G. Mendelian randomization: genetic anchors for causal inference in epidemiological studies. *Hum Mol Genet*. 2014;23(R1):R89-R98.
9. Smith GD. Use of genetic markers and gene-diet interactions for interrogating population-level causal influences of diet on health. *Genes & nutrition*. 2011;6(1):27-43.
10. Smith GD, Ebrahim S. 'Mendelian randomization': can genetic epidemiology contribute to understanding environmental determinants of disease? *Int J Epidemiol*. Feb 2003;32(1):1-22.
11. Davies NM, Holmes MV, Smith GD. Reading Mendelian randomisation studies: a guide, glossary, and checklist for clinicians. *BMJ*. 2018;362
12. Skrivankova VW, Richmond RC, Woolf BAR, et al. Strengthening the reporting of observational studies in epidemiology using mendelian randomisation (STROBE-MR): explanation and elaboration. *BMJ*. 2021;375:n2233. doi:10.1136/bmj.n2233
13. Lee JJ, Wedow R, Okbay A, et al. Gene discovery and polygenic prediction from a genome-wide association study of educational attainment in 1.1 million individuals. *Nat Genet*. Jul 23 2018;50(8):1112-1121. doi:10.1038/s41588-018-0147-3
14. Zhou H, Kember RL, Deak JD, et al. Multi-ancestry study of the genetics of problematic alcohol use in over 1 million individuals. *Nature Medicine*. 2023/12/01 2023;29(12):3184-3192. doi:10.1038/s41591-023-02653-5
15. Kurki MI, Karjalainen J, Palta P, et al. FinnGen provides genetic insights from a well-phenotyped isolated population. *Nature*. 2023/01/01 2023;613(7944):508-518. doi:10.1038/s41586-022-05473-8

© 2025 Capusan AJ et al. *JAMA Psychiatry*.

16. Saunders GRB, Wang X, Chen F, et al. Genetic diversity fuels gene discovery for tobacco and alcohol use. *Nature*. 2022/12/01 2022;612(7941):720-724. doi:10.1038/s41586-022-05477-4
17. Okbay A, Wu Y, Wang N, et al. Polygenic prediction of educational attainment within and between families from genome-wide association analyses in 3 million individuals. *Nature Genetics*. 2022/04/01 2022;54(4):437-449. doi:10.1038/s41588-022-01016-z
18. Demange PA, Malanchini M, Mallard TT, et al. Investigating the genetic architecture of noncognitive skills using GWAS-by-subtraction. *Nature Genetics*. 2021/01/01 2021;53(1):35-44. doi:10.1038/s41588-020-00754-2
19. Trubetskoy V, Pardiñas AF, Qi T, et al. Mapping genomic loci implicates genes and synaptic biology in schizophrenia. *Nature*. 2022/04/01 2022;604(7906):502-508. doi:10.1038/s41586-022-04434-5
20. Als TD, Kurki MI, Grove J, et al. Depression pathophysiology, risk prediction of recurrence and comorbid psychiatric disorders using genome-wide analyses. *Nature Medicine*. 2023/07/01 2023;29(7):1832-1844. doi:10.1038/s41591-023-02352-1
21. Howard DM, Adams MJ, Clarke T-K, et al. Genome-wide meta-analysis of depression identifies 102 independent variants and highlights the importance of the prefrontal brain regions. *Nat Neurosci*. 2019;22(3):343-352.
22. Demontis D, Walters GB, Athanasiadis G, et al. Genome-wide analyses of ADHD identify 27 risk loci, refine the genetic architecture and implicate several cognitive domains. *Nature Genetics*. 2023/02/01 2023;55(2):198-208. doi:10.1038/s41588-022-01285-8
23. Rosoff DB, Clarke T-K, Adams MJ, et al. Educational attainment impacts drinking behaviors and risk for alcohol dependence: results from a two-sample Mendelian randomization study with ~780,000 participants. *Mol Psychiatry*. 2021/04/01 2021;26(4):1119-1132. doi:10.1038/s41380-019-0535-9
24. Sanderson E. Multivariable Mendelian Randomization and Mediation. *Cold Spring Harb Perspect Med*. Feb 1 2021;11(2)doi:10.1101/cshperspect.a038984
25. Sanderson E, Glymour MM, Holmes MV, et al. Mendelian randomization. *Nature Reviews Methods Primers*. 2022/02/10 2022;2(1):6. doi:10.1038/s43586-021-00092-5
26. Sanderson E, Davey Smith G, Windmeijer F, Bowden J. An examination of multivariable Mendelian randomization in the single-sample and two-sample summary data settings. *Int J Epidemiol*. 2018;48(3):713-727. doi:10.1093/ije/dyy262

© 2025 Capusan AJ et al. *JAMA Psychiatry*.
