# Supplementary material for: Measures of General Intelligence and Risk for Alcohol Use Disorder
Source: JAMA Psychiatry. 2025 Oct 1;82(12):1195–202. doi: 10.1001/jamapsychiatry.2025.2689 (PMC12489797; doi:10.1001/jamapsychiatry.2025.2689)
Supplement: Supplement 3. — Data Sharing Statement. [file jamapsychiatry-e252689-s003.pdf]

Data Sharing Statement

Capusan. Measures of General Intelligence and Risk for Alcohol Use Disorder. *JAMA Psychiatry*. Published October 01, 2025. doi:10.1001/jamapsychiatry.2025.2689

Data
Data available: No

Additional Information
Explanation for why data not available: Swedish register data would require a separate ethics approval. GWAS summary statistics used are in the publica domain.
